# Utilizing mechatronic agilent gas chromatography to validate therapeutic efficacy of *Combretum paniculatum* against oxidative stress and inflammation

**DOI:** 10.1016/j.heliyon.2024.e36586

**Published:** 2024-08-22

**Authors:** Ifeoma F. Chukwuma, Kennedy Chinedu Okafor, Victor O. Apeh, Florence N. Nworah, Chigozie Paul Odo, Ijeoma Peace Okafor, Kelvin Anoh, Okoronkwo Chukwunenye Anthony

**Affiliations:** a Department of Biochemistry, Faculty of Biological Sciences, University of Nigeria, Enugu State, 410001, Nigeria; b Department of Genetics and Biotechnology, Faculty of Biological Sciences, University of Nigeria, Enugu State, 410001, Nigeria; c Department of Engineering, Faculty of Science and Engineering, Manchester Metropolitan University, M1 5GD, Manchester, UK; d Department of Electrical and Electronic Engineering Science, University of Johannesburg, Johannesburg, 2006, South Africa; e Cardiff Metropolitan University, Department of Applied Public Health, Cardiff, CF5 2YB, UK; f Department of Mechatronics Engineering, School of Electrical Systems Engineering and Technology (SESET), Federal University of Technology Owerri, 1526, Nigeria; g Department of Applied Sciences, Federal University of Allied Health Sciences, Enugu State, 01473, Nigeria; h School of Engineering, University of Chichester, Bognor Regis, PO21 1HR, UK

**Keywords:** Artificial intelligence, Biomedical ADMET properties, Biomedical molecular docking, Medical mechatronics, Mechatronic agilent gas chromatography, Bioactive compounds, Oxidative stress, Phytochemical composition

## Abstract

The quest for novel antioxidant and anti-inflammatory medications from medicinal plants is crucial since the plants contain bioactive compounds with a better efficacy and safety profile than orthodox therapy. This study harnesses the capabilities of mechatronics-driven Agilent Gas Chromatography, deploying *in vitro, in vivo*, and in silico models to unravel the antioxidant and anti-inflammatory attributes within *Combretum paniculatum* ethanol extract (CPEE). Employing gas chromatography-mass spectroscopy (GC-MS), our analysis efficiently segregates and evaluates volatile compound mixtures, a technique renowned for identifying organic compounds, as exemplified by its success in detecting fatty acids in food and resin acids in water. Using gas chromatography-mass spectrometry (GC-MS) and GC-FID analyses, this paper ascertains the comprehensive phytochemical composition of CPEE. Also, Molecular interactions of identified compounds with cyclooxygenase (COX-2) implicated in inflammatory urpsurge is verified. GC-MS and GC-FID analyses unveil 41 phytoconstituents within CPEE. Based on the *in vitro* research, CPEE demonstrated potential in inhibiting thiobarbituric acid-reactive substances, nitric oxide, and phospholipase lipase A2 with inhibition rates of 2.284, 6.547, and 66.8 μg/mL respectively. *In vivo* experiments confirm CPEE's efficacy in inhibiting granuloma tissue formation, lipid peroxidation, and neutrophil counts compared to untreated rats. Moreover, CPEE elicited a significant (P < 0.05) increase in the activities of SOD, CAT, and GSH concentrations while decreasing C-reactive protein, signifying promising therapeutic potential. Highlighting interactions between top-scoring phytoligands (epicatechin, catechin, and kaempferol) and COX-2, the findings underscore their drug-like characteristics, favorable pharmacokinetics, and enhanced safety toxicity profiles. Results from *in vitro, in vivo*, and in silico studies, highlights CPEE remarkable antioxidant and anti-inflammatory potentials.

## 1 Introduction

The inflammatory cascade is initiated as part of physiological homeostasis to eliminate infectious and non-infectious stimuli and begin a self-limiting healing process [1]. However, an abrogated inflammatory response and its associated diseases ranging from cancer, diabetes, and obesity to cardiovascular diseases [2] threaten public health worldwide [3], decreasing both life expectancy and quality of life [2]. Epidemiological data have shown that three of every five individual deaths result from chronic inflammatory-induced diseases [2]. Sadly, the incidence of these diseases is anticipated to rise exponentially over the next 30 years [2]. The root cause of cellular damage under chronic inflammation is derangement in the activation and release pro-inflammatory cytokines and mediators. This provokes organ damage, cell death, or cell stimulation, initiating excessive generation of reactive species leading to oxidative stress. In this framework, oxidative stress regulation is fundamental to human health preservation and a powerful option for preventing and treating important pathologies [4].

Several anti-inflammatory protocols, from non-steroidal anti-inflammatory drugs (NSAIDs), corticosteroids, metformin to other immunosuppressive therapies, are prescribed to alleviate the inflammatory response [1,5]. Unfortunately, these therapies are mostly disease-modifying, release symptoms transiently, and also induce life-threatening damaging effects on the liver, kidney, heart, and gastrointestinal tract after chronic usage [1,6,7]. Consequently, there is a great demand for safer and better alternative remedies. The inroad for the discovery of natural antioxidant and anti-inflammatory agents is mainly because of their safety profile, biocompatibility, high availability, and multi-targeted therapeutic efficacy in modulating several diseases including inflammation [7].

Over the past decades, phytocompounds have been engineered to enhance their therapeutic characteristics with newer technology. Thus, using in silico techniques in drug design and development has deepened the understanding of the detailed molecular interactions of phytocompounds with receptors, enzymes, and proteins, implicated in pathology of human diseases [8]. *Combretum paniculatum,* commonly called “forest frame”, is a valuable medicinal plant with established pharmaceutical efficacy, including anti-viral, anti-cancer, antiulcer, anti-diarrhoea, and antioxidant activities [9]. It is classified in the Combretaceae family and is highly endemic in tropical Africa [10]. In the realm of ethnomedicine, *C. paniculatum* is employed to address disease conditions like diarrhoea, vomiting, chronic dysentery, stomach pain, leprosy, stomatitis, enlarged liver, and gonorrhoea [9,10]. However, there is still paucity of information on the potential active volatile and non-volatile components against COX-2, a key player in the inflammatory pathway which could be repurposed as a lead and effective anti-inflammatory remedy. Thus, our research aimed to validate the antioxidant and anti-inflammatory effects of CPEE using standard *in vitro* and *in vivo* approaches.

A comprehensive compound profiling of CPEE were for the first time done with the aid of GC-MS and GC-FID to establish the link between the chemical constituents and biological activities. Furthermore, the identified phyto ligands were subjected to in silico studies to elucidate their molecular interactions with COX-2. The top-scoring compounds' drug-likeness, pharmacokinetic properties, and toxicity profile were also appraised.

## 2 Materials and methods

### 2.1 Chemicals and reagents

All the reagents and chemicals used in this research were of analytical grade and procured from reputable companies: British Drug Houses (BDH) Chemicals Ltd. Poole, England, Sigma‒Aldrich Inc., U.K. and Sisco Research Lab., India.

### 2.2 Preparation of the extract

Plant taxonomist at the International Centre for Ethnomedicine and Drug Development (InterCEDD) in Nsukka, Enugu State, Nigeria, collected and confirmed fresh leaves of *C. paniculatum*. The herbarium has a reference specimen of the leaf (INTERCEDD/1909). The shade-dried pulverised leaves were extracted using 2 L of ethanol-distilled water (70:30 v/v) for three days with occasional shaking. Subsequently, the macerate was then concentrated in a rotary evaporator set at 45 °C after being filtered first through Whatman No. 1 filters paper and then through muslin cloth. Thereafter, dried, *Combretum paniculatum* ethanol extract designated as CPEE, was kept in the refrigerator (4 °C) in a screw-capped container until needed.

### 2.3 CPEE phytochemical analysis

In this study, a Mechatronic Agilent Gas Chromatography (GC) system testbed was employed. This provides reliability, elevated sample throughput, and integrated Instrument Intelligence. The system provided analytical performance and operational efficiency through the embedding of intelligent predictive technologies, ensuring consistent uptime. In the paper, preliminary phytochemical screening of the CPEE was conducted to quantify the amounts of biologically important secondary metabolites. All tests followed the standard procedures of Harborne [11]and Trease & Evans [12]. The GC-MS and GC-FID analyses of CPEE were performed per the protocol outlined in Chukwuma et al. [13] using the Agilent GC-M910 model (Buck Scientific, Santa Clara, CA, USA). Based on the peak area created in the chromatogram, the relative concentrations of each phytochemical present were represented as a percentage. Compounds were identified by utilizing the GC retention time (RT) on the HP-5MS column and comparing the spectra with computer software standards (Replib and Mainlab data of GC-MS systems). For GC-FID screening, the ratio of the detected phytochemicals' area to the mass area of the internal standard, measured in μg/g, assisted in determining the phytochemicals.

### 2.4 Antioxidant and anti-inflammatory studies using *in vitro* models

Extent of lipid peroxidation, nitric oxide, and phospholipase A2 inhibitory effects of CPEE were investigated with conventional methods of Banerjee et al. [14] Sreejayan and Rao [15] and Vane [16], respectively with slight modifications. The standard drugs employed for the assays were butylated hydroxytoluene (BHT), ascorbic acid, and prednisolone, respectively.

### 2.5 Induction and treatment of chronic inflammation

The cotton pellet chronic inflammatory model was investigated based on the experimental protocol of Mosquera et al. [17]. Wistar rats (25) grouped randomly into five, each containing five rats. (n = 5). Accurately weighted, 20 mg of sterilised cotton pellet was implanted on both sides of the scapular regions of rats in groups 2–5. Subsequently, the rats were given distilled water, 100 mg/kg b.w. of diclofenac sodium (standard drug), CPEE, (100 and 200 mg/kg b.w.), respectively, orally for seven days, and on the eighth day, the rats were anaesthetized and blood samples were collected via cardiac puncture from all the rats before removing the pellet.

### 2.6 Assay of biochemical parameters

The blood samples collected from each rat was centrifuged at the speed of 604×*g* for 15 min. The sera were used to determine the following parameters: The extent of lipid peroxidation was investigated by measuring the concentration of malondialdehyde (MDA), a lipid peroxidation product, according to the method of Wallin et al. [18]. The endogenous antioxidants, (SOD and CAT) activities were measured in the sera following the experimental protocols of Fridovich [19] and Aebi [20], respectively. Concentration of glutathione (GSH) was measured with the Beutler [21] method while C-reactive protein (CRP) concentration was determined with a CRP ELISA kit (Abcam, Cambridge, MA, USA).

### 2.7 Molecular docking analysis

We consider docking as a technique used in molecular modeling to forecasts a molecule's preferred orientation in relation to another when a ligand and a target are fused to derive a stable-complex. With scoring functions, it is feasible to anticipate the strength of association or binding affinity between two molecules based on their knowledge preferred orientation [8]. By simulating molecular geometry and intermolecular forces and relying on stoichiometry and other fields of medical mechatronics, molecular docking technology can identify and anticipate the shape of receptor-ligand complexes. It highlights the "lock-&-key principle" of how ligands and receptors interact can be used to describe the mechanism of molecular docking.

In this paper, the target protein, cyclooxygenase-2 (PDB ID: 5IKQ), was prepared using the protein preparation wizard of the Schrodinger Suite v12.5 after being downloaded from the Protein Data Bank (PDB). The 3D conformers of all the phytochemicals identified with GC-MS and GC-FID and standard drug (diclofenac sodium) gotten from the PubChem database (https://pubchem.ncbi.nlm.nih.gov/) in SDF format were prepared following the procedure of Schrodinger Suite v12.05 LigPrep tools. Subsequently, the prepared ligands were then docked using standard precision (SP) and extra precision (XP) at the most predicted binding site of COX-2 using the Schrodinger Suite v12.05 glide docking tool. Additionally, the hit compounds' 2D and 3D interactions—which were chosen in accordance to the X-glide score—were also investigated.

### 2.8 Screening of drug-likeness, pharmacokinetics, and toxicity properties

Adsorption, Distribution, Metabolism, and Excretion (ADME) screening of the hits was predicted via the web server of SwissADME (https://www.swissadme.ch/). At the same time, toxicity prediction was made using the ProTox-II webserver (ProTox-II: Prediction of TOXicity of chemicals, https://tox-new.charite.de/protox_II).

### 2.9 Data analysis

Graph Pad Prism version 6.05 one-way ANOVA and Turkey's post hoc multiple comparisons were used to analyse the raw data set. The data were presented as mean ± standard deviation (SD) with significant thresholds of *p < 0.05, **p < 0.001, and ***p < 0.001. Graph Pad Prism's nonlinear regression curves estimated the half maximum inhibitory concentration (IC_50_) and R square (R^2^) values for our *in vitro* results.

## 3 Results

### 3.1 Phytochemical contents of CPEE

**Fig. 1:**
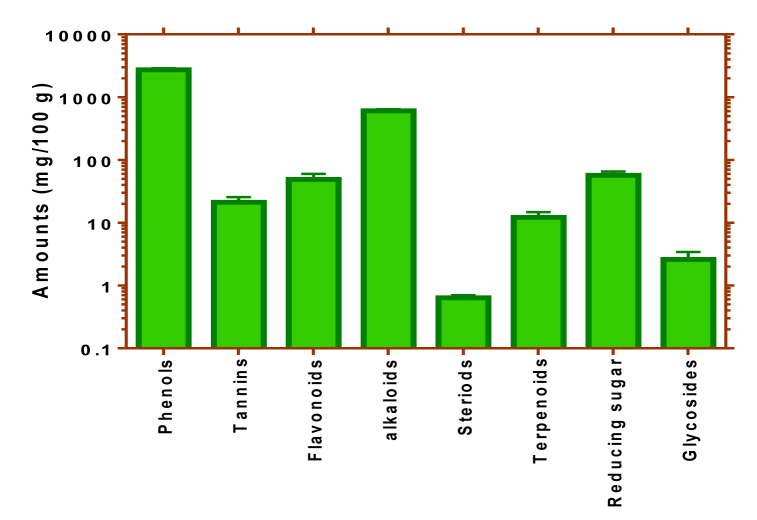
Preliminary phytochemical screening of CPEE. The values depict mean ± SD of triplicate data.

The results of preliminary phytochemicals screening showed that phenols, alkaloids, reducing sugar, and flavonoids were the four most prevalent compounds in CPEE. Moderate amounts of tannins and terpenoids were recorded, while glycosides and steroids were in low abundance (Fig. 1).

### 3.2 Compounds identified in CPEE using GC-MS and GC-FID

**Fig. 2:**
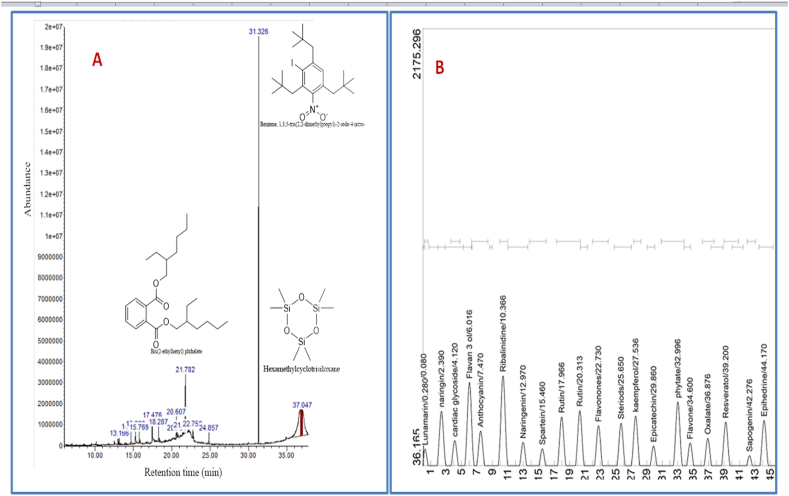
Chromatograms of compounds identified with GC-MS (A) and GC-FID (B).

The results of GC-MS indicated the presence of twenty-one compounds with decamethyltetrasiloxane, benzene,1,3,5-tris (2,2-dimethyl propyl)-2-iodo-4-nitro-, hexamethylcyclotrisiloxane, bis(2-ethylhexyl) phthalate and bis(trimethylsilyl) diethyl silicate as the prime components. N-Hexadecanoic acid, squalene, and 9-octadecenoic acid, together with thirteen other compounds, were also present (Table 1 and Fig. 2A). Similarly, GC-FID revealed the presence of twenty compounds that belong predominantly to flavonoids (proanthocyanidin, naringin, flavan-3-ol, anthocyanin, naringenin, rutin, flavanones, kaempferol, epicatechin, flavone, and resveratrol) and alkaloids classes (ribalinidine, sparteine, and ephedrine) (Table 2 and Fig. 2B).

**Table 1:** Phytochemicals identified in CPEE with GC-MS.

Peaks	Identified compound	M. WT (g/mol)	MF	RT	Area (%)
1	1-Octadecene	252.5	C_18_H_36_	13.018	0.53
2	Nonadecane	268.5	C_19_H_40_	13.166	0.63
3	Dibutyl phthalate	278.34	C_16_H_22_O_4_	14.704	1.38
4	n-Hexadecanoic acid	256.42	C_16_H_32_O_2_	15.303	2.10
5	5-Eicosene, (E)-	280.5	C_20_H_40_	15.769	1.19
6	9-Octadecenoic acid	282.5	C_18_H_34_O_2_	17.476	2.67
7	2-Methyl-1-hexadecanol	256.5	C_17_H_36_O	18.287	1.52
8	1-Docosene	308.6	C_22_H_44_	20.607	2.35
9	Octadecane	254.5	C_18_H_38_	20.701	0.60
10	Undecane, 4-cyclohexyl-	238.5	C_17_H_34_	21.515	0.41
11	Bis(2-ethylhexyl) phthalate	390.6	C_24_H_38_O_4_	21.782	6.22
12	Z-5-Nonadecene	266.5	C_19_H_38_	22.750	0.94
13	Squalene	410.7	C_30_H_50_	24.857	1.45
14	Benzene, 1,3,5-tris(2,2-dimethylpropyl)-2-iodo-4-nitro-	459.4	C_21_H_34_INO_2_	31.326	21.78
15	Decamethyltetrasiloxane	310.68	C_10_H_30_O_3_Si_4_	36.754	23.77
16	1,1,1,3,5,5,5-Heptamethyltrisiloxane	221.5	C_7_H_21_O_2_Si_3_	36.802	1.85
17	1,2-Bis(trimethylsilyl)benzene	222.47	C_12_H_22_Si_2_	36.836	1.73
18	Tris(tert-butyldimethylsilyloxy)arsane	468.7	C_18_H_45_AsO_3_Si_3_	36.880	2.94
19	Bis(trimethylsilyl) diethyl silicate	296.58	C_10_H_28_O_4_Si_3_	36.928	5.79
20	1-Methyl-2-Phenylindole	207.27	C_15_H_13_N	37.016	2.45
21	Hexamethylcyclotrisiloxane	222.46	C_6_H_18_O_3_Si_3_	37.047	17.69

**Table 2:** Phytochemicals identified in CPEE with GC-FID.

Peak no	Identified Compounds	Retention time (min.)	Peak Area	Peak Height	Conc. (μg/g)	Class of compounds
1	Proanthocyanidin	0.080	232.8066	139.940	0.2183	Flavonoid
2	Lunamarin	0.280	3400.3980	117.455	4.9786	Glycoside
3	Naringin	2.390	12252.8106	301.042	15.2487	Flavonoid
4	Cardiac glycoside	4.120	6344.5478	157.568	3.9370	Steroid
5	Flavan-3-ol	6.016	18154.0688	442.689	10.5455	Flavonoid
6	Anthocyanin	7.470	8442.9838	206.428	7.2410	Flavonoid
7	Ribalinidine	10.366	19598.0668	476.646	8.4040	Alkaloid
8	Naringenin	12.970	6238.3258	152.341	2.6361	Flavonoid
9	Sparteine	15.460	4967.5639	121.273	8.9024	Alkaloid
10	Rutin	20.313	12756.4840	307.630	7.9159	Flavonoid
11	Flavanones	22.730	9573.1408	233.186	8.2102	Flavonoid
12	Steriods	25.650	10008.8176	245.115	17.1678	Steroid
13	Kaempferol	27.536	11458.0104	280.295	5.2899	Flavonoid
14	Epicatechin	29.860	5478.4406	133.723	8.2188	Flavonoid
15	Phytate	32.996	14009.1853	345.703	18.8296	Anti-nutrient
16	Flavone	34.600	5756.4320	144.579	3.5721	Flavonoid
17	Oxalate	36.876	6988.5601	170.310	11.0380	Anti-nutrient
18	Resveratrol	39.200	10234.6024	249.263	7.7771	Flavonoid
19	Sapogenin	42.276	3473.1416	85.310	5.7077	Saponin
20	Ephedrine	44.170	10509.6768	256.782	13.5202	Alkaloid

### 3.3 Effects of CPEE on antioxidant and anti-inflammatory activities using *in vitro* models

**Fig. 3:**
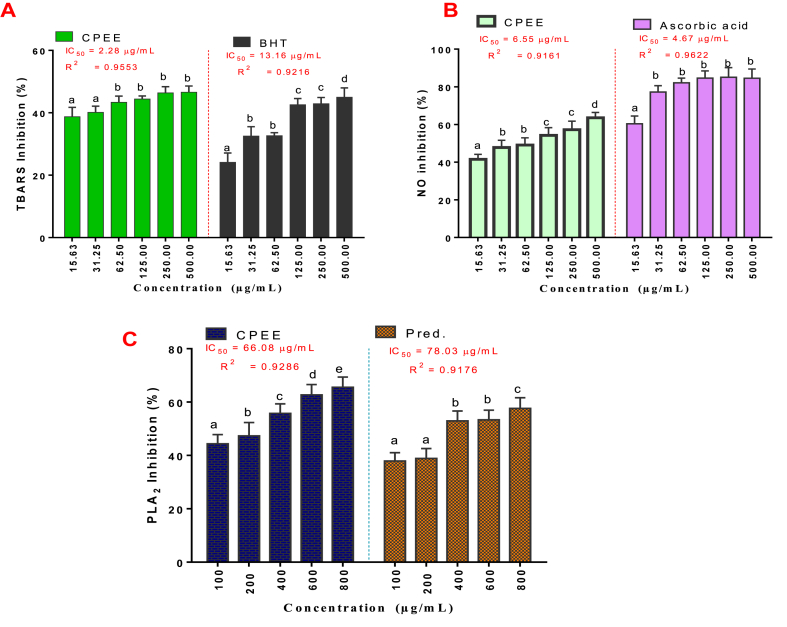
Effects of CPEE on TBARS (A), NO (B), and PLA2 (C) activities. Values depict mean ± SD of triplicate data.

The *in vitro* antioxidant and anti-inflammatory studies revealed that both CPEE and standard drug, BHT inhibited lipid peroxidation in a concentration-dependent manner, with maximal inhibition recorded at 500 μg/mL and IC_50_ values of 2.284 and 13.16 μg/mL, respectively (Fig. 3A). The extract also inhibited NO and PLA2 activities with IC_50_ of 6.547 and 66.08 μg/mL, respectively, compared with 4.667 and 78.03 μg/mL IC_50_ values obtained in the standard drugs, ascorbic acid and prednisolone, respectively (Fig. 3B and C).

### 3.4 Effects of CPEE on the weight of wet and dry granuloma tissue

The results in Table 3 show that treatment with 100 and 200 mg/kg of CPEE led to a dose-dependent, significant (p < 0.05) reduction in wet and dry granuloma tissue weight compared with the untreated group. The granuloma weight of the group treated with the standard anti-inflammatory drug, diclofenac sodium, was comparable higher than that of groups 3 and 4 treated with CPEE.

**Table 3:** Effects of CPEE on the weight of wet and dry granuloma tissue.

Experimental groups	Granuloma wet weight (mg)	Granuloma Dry weight (mg)	Δ in weight of cotton pellet (mg)
1	775.00 ± 21.21	239.00 ± 15.56	536.00 ± 5.66
2	498.00 ± 7.07***	177.00 ± 9.89*	321.00 ± 16.97**
3	430.00 ± 28.08****	150.00 ± 14.14**	280.00 ± 42.46***
4	400.00 ± 14.12****	138.00 ± 11.31**	262.00 ± 25.46***

The values depict mean ± SD (n = 5) with significant thresholds of *p < 0.05, **p < 0.001, and ***p < 0.00. Group 1 was not implanted with cotton pellet or treated (baseline) while cotton pellets were implanted to rats in groups 2–5, after which they were administered distilled water, 100 mg/kg b. w. diclofenac sodium, CPEE, (100 and 200 mg/kg. b. w), respectively.

### 3.5 Effects of CPEE on MDA and antioxidant markers of rats implanted with a cotton pellet

**Fig. 4:**
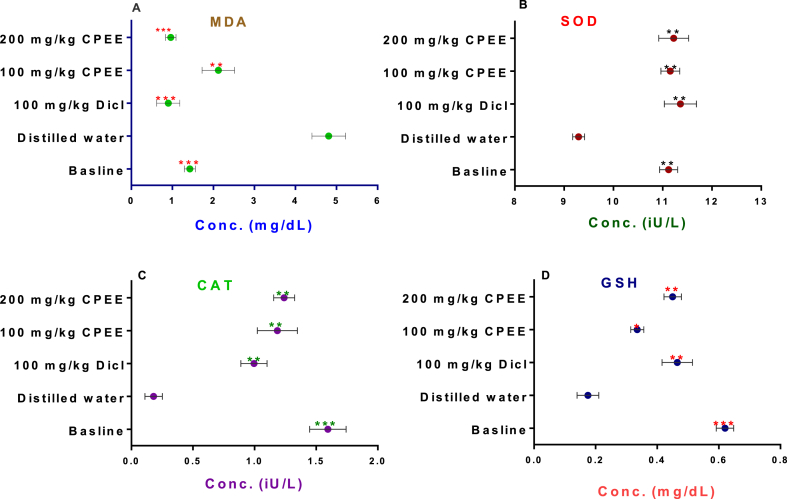
Effects of CPEE on MDA (A), SOD (B), CAT (C) and GSH (D) of rats implanted with a cotton pellet. The values depict mean ± SD (n = 5) with significant thresholds of *p < 0.05, **p < 0.001, and ***p < 0.001. Abbreviations: Malondialdehyde (MDA), superoxide dismutase (SOD), catalase (CAT) and reduced glutathione (GSH).

Cotton pellet-induced chronic inflammation elicited a remarkable increase in the concentrations of MDA and a reduction in SOD, and CAT activities, and concentration of GSH in the untreated rats relative to group 1 (baseline). Interestingly, groups treated with diclofenac sodium and varied doses of CPEE attenuated these anomalies by decreasing MDA concentration (Fig. 4A) while elevating the activities of SOD (Fig. 4B) and CAT (Fig. 4C) together with the concentration of GSH (Fig. 4D) relative to the untreated rats.

### 3.6 Effects of CPEE on C-reactive protein of rats implanted with cotton pellet

**Fig. 5:**
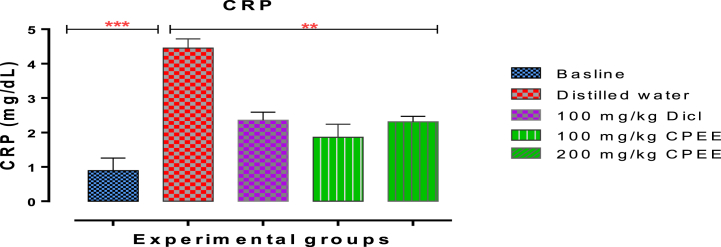
Effects of CPEE on C-reactive protein concentration of rats implanted with cotton pellet. The values depict mean ± SD (n = 5) with significant thresholds of *p < 0.05, **p < 0.001, and ***p < 0.00. Group 1 was not implanted with cotton pellet or treated (baseline) while cotton pellets were implanted to rats in groups 2–5, after which they were administered distilled water, 100 mg/kg b. w. diclofenac sodium, CPEE, (100 and 200 mg/kg b. w), respectively.

We realised that the concentration of CRP elevated significantly (p < 0.05) in the untreated group relative to the baseline not-implanted cotton pellet. However, treatment with the graded doses of CPEE and diclofenac sodium resulted in a remarkable decline in CRP concentration (Fig. 5).

### 3.7 Effects of CPEE on *in vivo* leukocyte mobilization

Results in Table 4 show that 100 and 200 mg/kg of CPEE administration inhibited neutrophil mobilization compared with group 2. However, the reverse was the case with lymphocytes, which decreased in the untreated group. Neutrophils and lymphocytes were the most mobilised leukocyte types; however, more neutrophils than lymphocytes were reported to be mobilised.

**Table 4:** Effects of CPEE on leukocyte mobilization.

Experimental groups	Differential leukocyte counts (%)				
	N	L	E	M	B
**1**	56.67 ± 3.06	42.67 ± 4.16	0.67 ± 0.15	0.00	0.00
**2**	68.00 ± 2.83	30.00 ± 2.83	2.00 ± 0.00	0.00	0.00
**3**	61.00 ± 4.24	38.00 ± 5.66	1.00 ± 0.14	0.00	0.00
**4**	56.00 ± 0.00	44.00 ± 0.00	0.00 ± 0.00	0.00	0.00
**5**	58.00 ± 8.48	39.00 ± 8.49	1.00 ± 0.00	0.00	0.00

The values depict mean ± SD (n = 5) with significant thresholds of *p < 0.05, **p < 0.001, and ***p < 0.00. Group 1 was not implanted with cotton pellet or treated (baseline) while cotton pellets were implanted to rats in groups 2–5, after which they were administered distilled water, 100 mg/kg b. w. diclofenac sodium, CPEE, (100 and 200 mg/kg b. w), respectively. N, L, E, M, and B stands for neutrophils, lymphocytes, eosinophils, monocytes and basophils, respectively.

### 3.8 Molecular interactions of CPEE phytoligands with COX-2

**Fig. 6:**
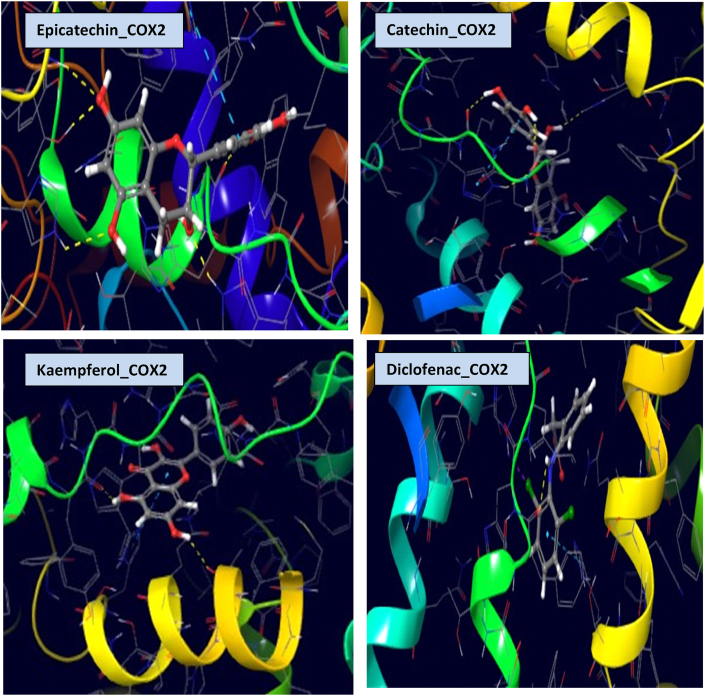
3D view of molecular interactions of epicatechin, catechin, kaempferol and diclofenac sodium with COX-2.

**Fig. 7:**
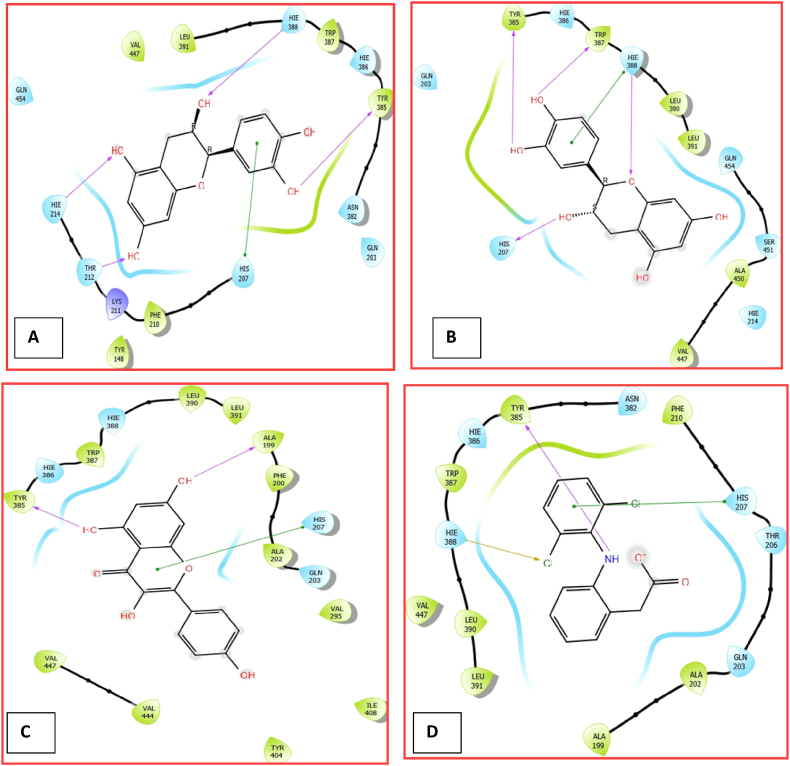
2D view of molecular interactions of epicatechin (A), catechin (B), kaempferol (C), and diclofenac sodium (D) with COX-2.

The molecular docking results in Table 5 showed that the three top prioritized compounds (epicatechin, catechin, and kaempferol) bound favorably to the binding cavities of COX-2 with binding energies of −8.618, −8.336, and −8.096 kcal/mol, respectively against −5.332 kcal/mol recorded in diclofenac sodium. The docking complex of epicatechin with COX-2 was stabilised by four hydrogen bonds with THR 212, HIE 214, HIE 388, and TYR 385 and pi-pi stacking with HIS 207. Similarly, catechin had four hydrogen bond interactions with TYR 385, TRP 387, HIE 388, HIS 207, and a Pi-pi stacking with HIE 388. The two residues involved in hydrogen bonding with kaempferol were ALA 199 and TYR 385, while HIS 207 had pi-pi stacking with kaempferol. In addition, the diclofenac sodium-COX-2 complex was stabilised by a halogen bond (HIE 388), a hydrogen bond (TYR 385), and pi-pi stacking (HIS 207) (Table 5, Fig. 6, Fig. 7).

**Table 5:** Molecular interactions of CPEE phytoligands with COX-2.

Compounds	Interacting residue	Types of interaction	Distance (À)	Binding energy, (kcal/mol)	XP Glide scores (kcal/mol)
Epicatechin	THR 212	H. bonding	2.34	−8.618	−8.618
	HIE 214	H. bonding	2.76		
	HIE 388	H. bonding	1.88		
	TYR 385	H. bonding	1.89		
	HIS 207	Pi-pi stacking	5.06		
	TYR 385	H. bonding	2.04		
Catechin	TRP 387	H. bonding	2.00	−8.336	−8.336
	HIE 388	H. bonding	2.24		
	HIE 388	Pi-pi stacking	5.17		
	HIS 207	H. bonding	2.11		
	HIS 207	Pi-pi stacking	5.39		
Kaempferol	ALA 199	H. bonding	2.11	−8.096	−8.126
	TYR 385	H. bonding	1.86		
	HIE 388	Halogen bond	3.32		
Diclofenac Sodium	TYR 385	Hydrogen bond	2.64	−5.332	−5.332
	HIS 207	Pi-pi stacking	4.54

### 3.9 Drug-likeness, pharmacokinetics and toxicity properties

The three top-scoring compounds from CPEE (epicatechin, catechin, and kaempferol) had good physicochemical properties, zero violations of the Lipinski rule for available oral drugs having MW < 500, hydrogen bond acceptor <10, hydrogen bond donor <5, and LogPo/w < 5. The hits compounds had 0.55 bioavailability score, high gastrointestinal absorption (GIA), and lead-likeness properties and were not BBB. Diclofenac sodium shared similar features with the top scoring compounds except that it can permeate the BBB and was predicted to violate one of the rules for lead-likeness, having an XLOGP3 > 3.5 (Table 6).

**Table 6:** Drug-likeness, pharmacokinetics and toxicity properties.

Properties	Epicatechin	Catechin	Kaempferol	Diclofenac sodium	References
PubChem CID	72276	9064	5280863	5018304	–
Formula	C_15_H_14_O_6_	C_15_H_14_O_6_	C_15_H_10_O_6_	C_14_H_10_Cl_2_NNaO_2_	–
MW (g/mol)	290.27	290.27	286.24	318.13	150–500
R. bond	1	1	1	4	0–9
HB donor	5	5	4	1	01–5
HB acceptor	6	6	6	2	0–10
MR	74.33	74.33	76.01	75.61	40–130
TPSA	110.78	110.78	111.13	52–16	20–130
LogPo/w (XLOGP3)	0.36	0.36	1.90	4.4	−0.7–5
Consensus Log P	0.85	−0.56	1.58	0.65	≤3.5
Fraction Csp3	0.2	0.2	0.00	0.07	−0.25 - < 1
Drug-likeness (Lipinski rule)	Yes 0 violation	Yes 0 violation	Yes 0 violation	Yes 0 violation	MLog p ≤ 4.15,MW ≤ 500,HBA ≤10, HBD ≤5
Lead-likeness	Yes	Yes	Yes	No (XLOGP3 > 3.5)	250 ≤ M.W ≤ 350, XLOGP3 ≤ 3.5, R-bonds ≤7
BA score	0.55	0.55	0.55	0.55	>0.1 (10 %)
BBB	No	No	No	Yes	BBB^+^ ≥ 0.30, BBB^−^ < −1
GIA	High	High	High	High	
Synthetic Accessibility	3.5	3.60	3.14	2.23	1-10 (very easy –very difficult)
Log kp (cm/s)	−7.82	−7.82	−6.70	−5.12	- −8.0 to −1.0
Predicted LD_50_ (mg/kg)	10000	10000	3919	53	–
Toxicity class	6	6	5	3	–

**Keys:** MW; molecular weight, R. bond; rotatable bonds, HB; hydrogen bond, M.R; molar reflectivity, TPSA; topological polar surface area, BA. Score; bioavailability score, BBB; blood brain barrier GIA; gastrointestinal, LD_50_; lethal dose.

## 4 Discussion

Medicinal plants' antioxidant and anti-inflammatory actions are strongly correlated with the abundance of bioactive phytochemicals [22,23]. Our preliminary phytochemical screening revealed that CPEE contains rich amounts of phenols, tannins, flavonoids, alkaloids, reducing sugar, and other pharmaceutical-relevant compounds. Data from several research studies have established the antioxidant effects of phenolic compounds (phenols, flavonoids, and tannins) due to their hydrogen/electron-donating capacity from their multiple phenolic rings [23,24]. Phenols modulate inflammation and its associated complications by suppressing the expression of pro-inflammatory genes, which can provide some hints for inflammatory therapies using phenol intervention [25]. Compelling evidence shows that the remarkable anti-inflammatory action of flavonoids is via regulation of several signaling pathways involved in inflammation, such as MAPK, PI3K/Akt, NF-***κ***B, Nrf2, and STAT signaling pathways [24]. Leading credence to this statement, Miao et al. [24], reported that flavonoids can drastically regulate symptoms of rheumatoidd arthritis by inhibiting systemic and local inflammation responses. Additionally, tannins and alkaloids' antioxidant and anti-inflammatory effects have been registered in many experimental studies [26].

We subjected CPEE to GC-MS and GC-FID metabolite profiling to further substantiate the presence of pharmacologically relevant compounds. Among the compounds identified with GC-MS, 9-octadecenoic acid, hexadecanoic acid, and squalene have profound antioxidant and anti-inflammatory actions [27]. According to Bensaad et al. [28], octadecanoic acid exerts anti-inflammatory effects by either inhibiting prostaglandin (PG) release or biosynthesis processes possibly via blockage of cyclooxygenase activity or by directly acting on the hypothalamus to impede the production of pro-inflammatory agents. Likewise, quenching of singlet oxygen species and biomembrane stabilization are squalene's registered antioxidant and anti-inflammatory effects [27]. Hexamethylcyclotrisiloxane, affluent in CPEE, has been reported to be a potent antimicrobial, antibacterial, and antioxidant agent that is paramount for scavenging free radicals [29]. The antioxidant and anti-inflammatory activities of the compounds identified from GC-FID, including rutin, catechin, naringenin, kaempferol, anthocyanin, and epicatechin, have also been documented [13]. The richness of these compounds in CPEE could possibly define its biological action as a remarkable antioxidant and anti-inflammatory agent.

Reports from experimental and clinical diagnosis have buttressed the assertion that unregulated generations of ROS and RNS, such as nitric oxide in the inflammatory response, escalate the peroxidation of biomembranes, lipids, proteins, and DNA, leading to disruption of membrane integrity, proteins deamination, and fragmentation of DNA strands by endonucleases [23,29]. Although nitric oxide serves as a helpful signalling molecule, excessive NO combines with oxidant species to form peroxynitrite, a trigger of lipid peroxidation and an upregulator of COX-2 expression [23]. Interestingly, CPEE inhibited lipid peroxidation, NO, and PLA2 activity. The decrease in NO scavenging activity by CPEE suggests that it could ameliorate excessive NO, thereby preventing modification of cellular molecules. Compelling evidence has also shown that substances that inhibit NO and PLA2 activities are vital to impede the advancement of inflammation [5]. It has been established that inhibition of PLA2 could be achieved by either inhibiting PLA2 catalytic activity or release of its substrate, phospholipids from biomembranes [5]. Although the phytocompound responsible for this activity was not ascertained in the present studies, previous research has reported PLA2 inhibitory effects of some of our identified compounds [27]. In this context, the antioxidant, and anti-inflammatory activities of CPEE can be inferred from its TBARS, NO and PLA2 inhibitory properties, which indicate that it could serves as a rich source of natural antioxidant and anti-inflammatory agents.

The cotton pellet is an inducing agent of inflammation that stimulates cellular infiltration, vascular permeability, and the generation of granuloma tissues. Thus, it is widely used to investigate the extent of proliferation and exudation of inflammatory proteins and cells due to tissue degeneration [6]. Here, we observed a significant (P < 0.05) reduction in the weight of the wet and dry granuloma tissue in the treated groups. Undoubtedly, the reduction of granuloma tissue by CPEE could result from decreased deposition of granuloma-forming cells, including neutrophils, fibroblasts, and macrophages at the inflamed site [6]. This pharmaceutical action could be hinged on the rich bioactive compounds present in the CPEE. Specifically, studies have demonstrated that phenols and flavonoids rich in CPEE inhibit the expression of pro-inflammatory genes [30]. Ideally, plants with antioxidant effects are highly sought as anti-inflammatory agents since they avert tissue damage and other biochemical cascades involved in granuloma tissue formation.

Endogenous antioxidants such as SOD, CAT, and GSH form an integral part of body defence needed to protect the cells against oxidative stress. Sadly, excessive ROS and other free radicals generated by inflammatory reactions alter the redox equilibrium, thereby overwhelming the functionality and expressions of these antioxidants [31]. In the present study, a significant (p < 0.05) decline in the endogenous antioxidants (SOD, CAT, and GSH) was recorded in the untreated group relative to the baseline. It is noteworthy that groups treated with CPEE sustained the activities of SOD, CAT, and concentration of GSH with a corresponding decline in MDA. Moreso, CPEE ability to raise antioxidant markers suggests that it can keep the reductive balance stable, which makes it a possible antioxidant agent. Moreover, the decrease in MDA further strengthened the antioxidant capacity of CPEE. These findings corroborate reports of previous studies by Onikpani et al. [22], and Miao *et al.* [24], where natural antioxidants were used as a remedy for oxidative stress and inflammation. This therapeutic action could be attributed to the scavenging action of the identified antioxidant phytochemicals, such as flavonoids, phenols, and squalene known to neutralize free radicals and activate endogenous antioxidant expression [26,27,30].

C-reactive protein (CRP) mediates the acute-phase response in inflammation by activating complement and cell-mediated pathways, which release pro-inflammatory cytokines [32]. Studies have shown increased deposition of CRP at sites of tissue damage and inflammation in naturally occurring and experimental conditions [32]. Consistent with previous studies, we realised that the concentration of CRP in the untreated rats was significantly (p < 0:05) elevated compared with the baseline. The recorded reduction in CRP, a vital biomarker of both acute and chronic inflammation, after treatment with CPEE is a pointer to its anti-inflammatory action. Ideally, activation of NF-κB by inflammatory agents induces the expression and release of specific pro-inflammatory cytokines, including TNF-α, IL-6 and IL-8 which activates a number of acute-phase proteins, including CRP [33]. Besides CRP elevation, the first cellular response in inflammation begins with infiltrating leucocytes to the inflamed site to eliminate the foreign particle [34]. In this study, we recorded a decline in the neutrophils and an increase in lymphocytes after treatment with CPEE. Although neutrophils play a pivotal role in phagocytosis and eliminating pathogens, they enhance the production of reactive oxygen intermediates [35]. The ability of CPEE to reduce neutrophil migration supports its inherent anti-inflammatory potential, which will be useful in halting inflammatory progress. Compelling evidence has registered that the decrease in lymphocytes recorded in the untreated group is a reflection of nutritional and inflammatory cascade in the body. Thus lower lymphocytes are correlated to death of patients [36].

The molecular docking (MD) technique is an algorithm capable of predicting the interaction of druggable candidates with appropriate receptors or proteins with high accuracy, providing valuable insights into the mechanistic action of a drug [[37], [38], [39], [40]]. So, we used MD to figure out how CPEE bioactive compounds might interact with COX-2, which is involved in the inflammatory cascade. Interestingly, in collaboration with our *in vitro* and *in vivo* results, our in silico studies revealed that the CPEE epicatechin, catechin, and kaempferol docked very well to the binding cavity of COX-2 using hydrogen bonds, pi-pi stacking, and halogen bonds between the phenyl and alkyl residues. Compelling evidence has noted that hydrogen bonding is crucial for enzyme catalysis and the structural stability of biomolecules. Besides, interactions of amino acid residues with aromatic rings play a vital role in drug design since they improve molecular interactions and recognition, enhance drug specificity and therapeutic efficacy [8]. The modes of action displayed by our hits were similar to those of the standard COX-2 inhibitor, diclofenac sodium. According to Modak et al. [1], the COX-2 substrate binding site consists of ARG 120, TYR 385, and GLU 524. Specifically, COX-2 active site is occupied by HIS 207 which acts as proton acceptor and TYR 385 for cyclooxygenase activity while HIE 388 serves as metal ion binging site [41]. It is worth mentioning that all our hits interacted with HIS 207 and TYR 385 found in the active site of COX-2. Undoubtedly, binding to this active site amino acid residues and other useful amino acid residues of COX-2 will hinder binding of the actual substrate, arachidonic acid, thereby inhibiting the production of prostaglandin and ultimately translating to downregulation of the inflammatory reactions [22,31].

The high failure rate at the different stages of drug design and development has fostered computational models such as Absorption, Distribution, Metabolism, Elimination, Toxicity (ADMET) prediction [8], which can predict the drug-likeness of a drug candidate with high precision [39,42]. Consequently, agents with unfavourable ADMET qualities could be eliminated from the list of possible drug candidates early enough, thereby saving the cost of developing a non-marketable drug [38,43]. Lipinski's rule-of-five sheds light on the relationship between a compound's physicochemical properties and bioavailability [8,44,45]. Therefore, the pharmacological design of a drug molecule must start with excellent bioavailability since without it, the aim of administering a drug molecule into the body would be pointless [43]. Interestingly, all our hits (epicatechin, catechin, and kaempferol) passed drug-likeness rules. Also, the compounds had favorable bioavailability scores, high gastrointestinal absorption, and log Kp, making them effective oral drugs, which is highly preferred for the patient's compliance and comfort [44]. The hits also had lead-likeness against diclofenac sodium, which violated one of the rules by having high lipophilic value (XlogGP3 >3.5). The high lipophilic property of a drug candidate leads to increased metabolic turnover, minimal intestinal absorption, low solubility, and higher toxic impact on key organs [46]. Importantly, all the hits fit well within the safety profile range for humans since they were predicted to belong to the toxicity classes of 5–6 and have LD_50_ values in the range of 3919–10000 mg/kg body weight. In comparison, diclofenac sodium belongs to toxicity class 3 with an LD_50_ of 53 mg/kg b.w. Globally Harmonised System of Classification and Labelling of Chemicals standardization states that drugs in toxicity classes 3 with LD_50_ in the range of 50 < LD_50_ ≤ 300 are toxic if swallowed [47]. So, the toxicity prediction results show that our top-scoring compounds are safe while diclofenac could be toxic to humans. This means that CPEE phytoligands are effective and safe oral lead compounds for oxidative stress, inflammation, and their related complications.

## 5 Conclusions

This paper comprehensively explored the antioxidant and anti-inflammatory properties of medicinal plant extracts, specifically centering on the bioactive compounds present in *Combretum paniculatum* (CPEE). A multidisciplinary methodology was adopted, leveraging *in vitro, in vivo*, and in silico models. Additionally, advanced analytical techniques, including mechatronics-driven Agilent Gas Chromatography, are employed. The findings highlight the efficacy of CPEE as a natural source with potent antioxidant and anti-inflammatory agents, characterised by compounds such as phenols, flavonoids, and alkaloids. It was observed that these bioactive constituents exhibit notable potential in modulating key inflammatory pathways and inhibiting oxidative stress-induced damage. Throughout various analyses and experimental models, CPEE showcased a remarkable capacity to curtail granuloma tissue formation, uphold endogenous antioxidant levels, and diminish inflammatory biomarkers like CRP and neutrophils. Molecular docking studies further unveiled the ability of CPEE compounds to interact with COX-2, mirroring actions similar to standard anti-inflammatory drugs. Significantly, in silico ADMET and toxicity predictions endorse the drug-like qualities and safety profile of these compounds. This substantiates their promise for application in managing oxidative stress, inflammation, and associated ailments. This study unequivocally demonstrates the extraordinary antioxidant and anti-inflammatory potential of CPEE, largely attributed to its rich array of bioactive compounds. Consequently, it validates the utilisation of *C*. *paniculatum* in inflammation management and positions it as a primary candidate for the development of antioxidant and anti-inflammatory drugs. Future research aims to focus on predictive modeling for compound interactions, integrating Artificial Intelligence (AI) into medicinal plant research. This innovative approach promises to accelerate drug discovery and enhance therapeutic outcomes for inflammation-related conditions. Specifically, the investigation will explore an AI transform model designed for deriving antioxidant and anti-inflammatory drugs from medicinal plants, including applications in virtual screening for novel compounds. We are looking into AI-driven formulation design, route mapping, optimization of clinical trials, and extraction of ethnobotanical insights. This holistic approach holds the potential to revolutionize the field, expediting the development of effective treatments.

## Data availability

The authors do not have permission to share data.

## Ethical issues

The Ethics and Biosafety Committee of the Faculty of Biological Sciences, University of Nigeria, Nigeria approved the study (approval number: UNN/FBS/EC/1082).

## CRediT authorship contribution statement

**Ifeoma F. Chukwuma:** Writing – original draft, Validation, Methodology, Conceptualization. **Kennedy Chinedu Okafor:** Writing – review & editing, Project administration, Funding acquisition, Formal analysis. **Victor O. Apeh:** Writing – review & editing, Visualization, Methodology, Formal analysis, Data curation. **Florence N. Nworah:** Visualization, Software, Formal analysis, Data curation. **Chigozie Paul Odo:** Resources, Methodology, Investigation, Formal analysis, Conceptualization. **Ijeoma Peace Okafor:** Writing – review & editing, Visualization, Validation, Funding acquisition. **Kelvin Anoh:** Writing – review & editing, Visualization, Validation. **Okoronkwo Chukwunenye Anthony:** Validation, Resources, Project administration, Formal analysis.

## Declaration of competing interest

* None of the authors of this paper has a financial or personal relationship with other people or organizations that could inappropriately influence or bias the content of the paper.

* It is to specifically state that "No Competing interests are at stake and there is No Conflict of Interest" with other people or organizations that could inappropriately influence or bias the content of the paper.
